# Breaking the Bottleneck in Limit of Detection of Surface Refractive Index Sensing by Harnessing Meta‐Waveguide Microring Resonators

**DOI:** 10.1002/advs.75339

**Published:** 2026-04-21

**Authors:** Wanxin Li, Jiewen Li, Rui Li, Gina Jinna Chen, Perry Ping Shum, Yi Zou, Ray T. Chen, Yongkang Dong, Xiaochuan Xu

**Affiliations:** ^1^ National Key Laboratory of Laser Spatial Information Guangdong Provincial Key Laboratory of Integrated Photonic‐Electronic Chip Guangdong Provincial Key Laboratory of Aerospace Communication and Networking Technology Harbin Institute of Technology Harbin Institute of Technology campus Xili University Town Shenzhen Guangdong China; ^2^ State Key Laboratory of Optical Fiber and Cable Manufacture Technology Guangdong Key Laboratory of Integrated Optoelectronics Intellisense Department of EEE Southern University of Science and Technology Shenzhen China; ^3^ School of Information Science and Technology ShanghaiTech University Shanghai China; ^4^ Department of Electrical and Computer Engineering The University of Texas at Austin Austin Texas USA

**Keywords:** meta‐waveguide microrings, mode splitting, ultra‐sensitive self‐referenced sensing

## Abstract

Pushing sensor sensitivity to the extreme remains a central pursuit in sensing research. However, the limit of detection (LOD) is ultimately limited by the trade‐off between intrinsic sensitivity and noise: improvements in sensitivity often introduce higher noise, yielding only marginal gains in LOD. Here, we leverage mode splitting in meta‐waveguide microring resonators (MWMRs)—a typically overlooked phenomenon—to suppress drift‐induced bias and noise without sacrificing interfacial refractive‐index sensitivity. This approach reduces baseline drift to just 4.6 fm/min during representative measurements, enabling quantitative detection of ultra‐weak surface perturbations. The sensing performance is validated through the streptavidin‐biotin interaction, achieving reliable detection of 0.01 pg/mL streptavidin with a blank reference and a splitting noise of 1.3 pm. This reflection‐induced mode splitting framework breaks the longstanding LOD bottleneck in surface refractometric sensing and establishes MWMRs as a robust, drift‐immune platform for ultra‐sensitive optical interrogation.

## Graphical abstract

Mode splitting in meta‐waveguide microring resonators (MWMRs) effectively suppresses baseline drift and measurement noise while maintaining high refractive‐index sensitivity. This reflection‐induced mode splitting strategy enables quantitative detection of ultra‐weak surface perturbations and establishes MWMRs as a robust, drift‐immune platform for ultra‐sensitive optical sensing.

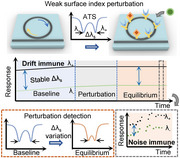

## 1 Introduction

Detecting analytes at ultra‐low concentrations is critical for applications including sub‐picomolar biomarker quantification, single‐pathogen identification, and contaminant monitoring in complex environments [1]. These demands have driven ongoing efforts to improve the limit of detection (LOD), defined as the lowest analyte concentration reliably measurable [2]. On‐chip microring resonators, characterized by their compact footprint, have emerged as promising sensing platforms [3, 4, 5, 6, 7, 8, 9]. However, their sensitivity is often insufficient as the majority of the optical field is confined within the waveguide core, resulting in weak interactions with surrounding analytes. Incorporating nanostructures into microrings, such as slot waveguides [10, 11], one‐dimensional photonic crystal waveguides [12, 13], and meta‐waveguides [14, 15, 16, 17], opens a new avenue to break the sensitivity limit imposed by conventional guided‐wave mechanisms. Despite significant advances in sensitivity, the improvement in LOD remains marginal, as sensitivity‐enhanced sensors generally increase susceptibility to environmental disturbances, frequently resulting in the deterioration of LOD. Therefore, overcoming the sensitivity‐noise constraint plays a key role in the detection of extremely refractive‐index perturbations. While the macroscopic noise, such as temperature fluctuations and vibrations, can be effectively suppressed by introducing reference channels [7, 18], noise arising from micro‐ or nanoscale regions remains difficult to mitigate and is particularly critical for detecting individual particles and surface binding dynamics.

In this work, we attempt to address this issue by exploring the mode splitting in meta‐waveguide microrings (MWMRs). Mode splitting typically arises from mutual coupling between clockwise (CW) and counterclockwise (CCW) modes in microrings, induced by reflective elements, such as sidewall roughness [19], scatterers [20, 21, 22], and periodic structures [23, 24, 25, 26, 27, 28], which lift mode degeneracy. It has been intensively investigated as a self‐referenced sensing architecture, demonstrating a remarkable ability to detect regional tiny refractive perturbations, such as a single particle or virus [20, 29] due to its inherent noise immunity. However, these approaches are not always beneficial for sensing performance (see Section S1). Accordingly, meta‐waveguides are adopted as a platform that enables controlled residual reflection while preserving low optical loss and strong field overlap with the sensing medium. While a meta‐waveguide is typically treated as a homogeneous uniaxial crystal [30] without reflection according to Effective Medium Theory (EMT), our recent findings reveal that this approximation is not universally applicable, particularly in resonant configurations [23]. In the practical finite periodic structure considered here, weak internal reflection arises from the finite‐length meta‐waveguide section (see Section S2 and Figure S1). In the microring, cavity loss and the finite *Q* factor further impose a finite effective interaction length, so that this internal reflection acts as the back‐coupling pathway between the CW and CCW modes, giving rise to mode splitting that can be leveraged for sensitive, low‐noise interrogation [31, 32, 33]. Building on this concept, we harness mode‐splitting‐based MWMRs for self‐referenced surface sensing. Interfacial refractive‐index variations modulate the internal reflection and consequently alter mode splitting, enabling quantitative and stable detection of ultra‐weak surface perturbations. This reflection‐induced mode splitting approach offers inherent stability and effectively decouples surface‐specific signals from environmental noise, positioning MWMRs as a robust photonic platform for high‐precision, surface‐selective sensing.

## 2 Results

### 2.1 The Origination of Mode Splitting in MWMRs

**FIGURE 1:**
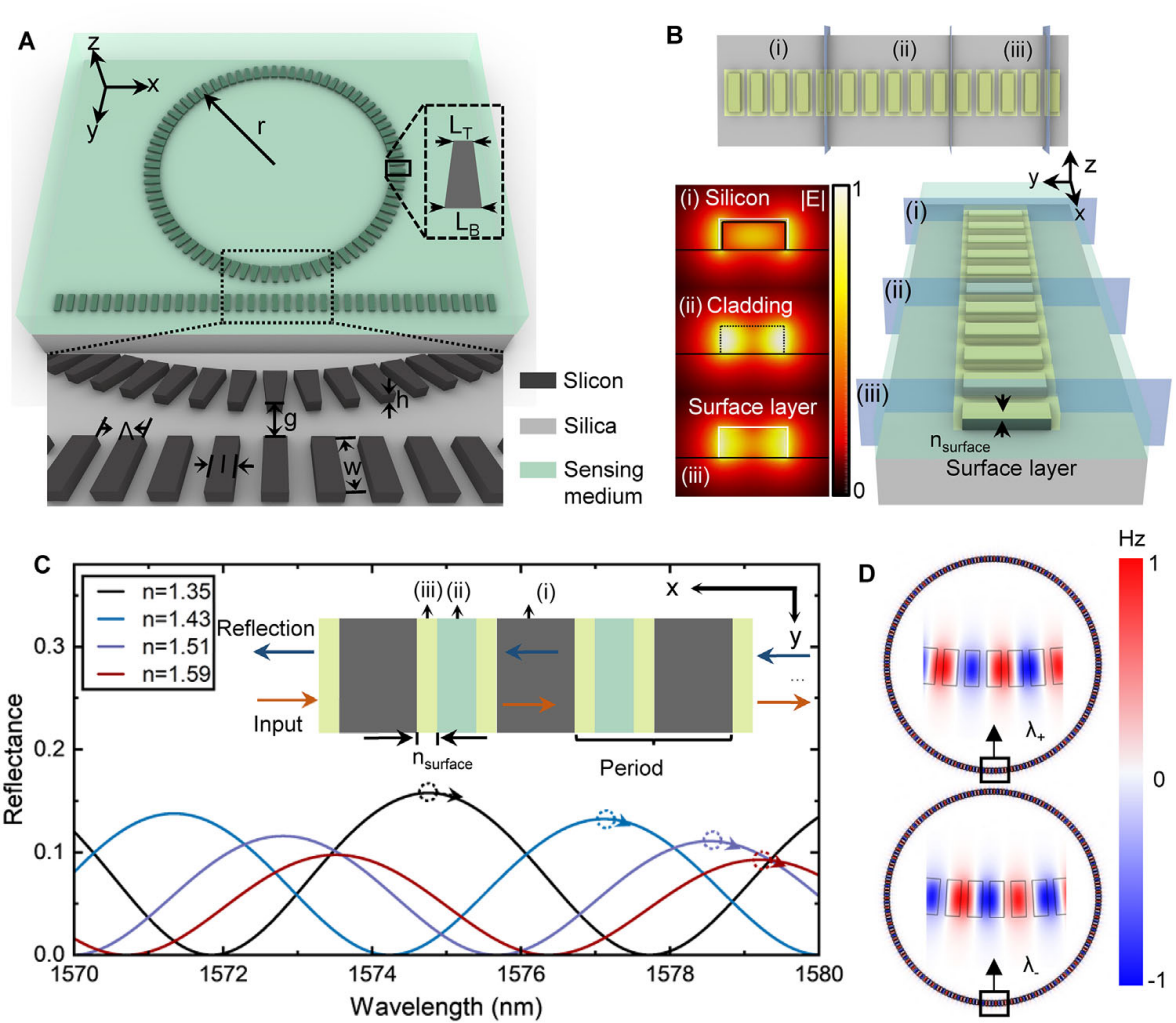
Structure and reflection origin of the MWMR. (A) Schematic of a meta‐waveguide side‐coupled to an MWMR, showing the side‐coupled configuration used for reflection modeling. Parameters of the meta‐structure: *L*_B_ = 240 nm, *L*_T_ = 160 nm, *r* = 10 µm, *g* = 500 nm, *h* = 220 nm, *w* = 500 nm, *l* = 180 nm. (B) Electric field distributions of the silicon (i), cladding (ii), and surface‐layer (iii) segments. Top inset: top‐view of the meta‐waveguide. (C) Red‐shifted reflectance spectra of the meta‐waveguide under varying surface‐layer refractive indices. Inset: three‐layer‐stack model used in reflection simulation. (D) Magnetic field (H_z_) distributions of the two split modes, with inset magnifying their distinct spatial profiles.

The proposed structure is a classic all‐pass MWMR, consisting of an MWMR side‐coupled to a meta bus waveguide, as shown in Figure 1A. The period (Λ) of the meta‐waveguide is 300 nm, which is sufficiently small to prevent diffraction (Λ < *λ*_0_/2*n*_eff_) [30]. The circularly arranged trapezoidal silicon pillars are adopted to minimize the intrinsic loss of the MWMR, while the meta bus waveguide is constructed with rectangular silicon pillars [16]. The gap (*g*) between the meta bus waveguide and the MWMR is optimized for critical coupling. In this configuration, the reflection response to interfacial refractive‐index variations is modeled using a three‐layer stack (silicon /cladding /surface layer, as shown in Figure 1B), with layer indices estimated by a field‐weighted effective‐index method [34]. The electric field distributions of the transverse‐electric (TE) mode of each layer are simulated by the finite element method (FEM) and depicted in the insets of Figure 1B. In practical sensing scenarios, the surface layer could be composite films such as functional coatings, protein probes, and blocking layers, as interfaces typically require sequential modifications before interacting with analytes. Without loss of generality, we model the surface film as a 10 nm‐thick uniform layer with refractive index (*n*) varying with material composition and molecular density [35]. In the simulation, the refractive index *n* is varied from 1.35 to 1.59, covering the range of typical organic and biomolecular films, including Bovine Serum Albumin (BSA, *n* = 1.47 [35]) and biotin–streptavidin complex (*n* = 1.502 [36]). Figure 1C shows the red‐shifted reflectance spectra (*R*) of the surface‐layer‐covered meta‐waveguide, obtained from a transfer‐matrix‐based equivalent‐reflection model of the finite periodic section (see Section S3 and Figure S2). The reflectance spectra are periodic functions of wavelength, with the peak reflectance reaching a maximum at *n* = 1.35 and gradually decreasing as the index contrast between materials diminishes. The residual reflection in the MWMR gives rise to mode splitting by lifting the degeneracy of CW/CCW modes. Consequently, Figure 1D presents the field distributions (H_z_) of the two split modes, with the inset magnifying their distinct spatial profiles.

**FIGURE 2:**
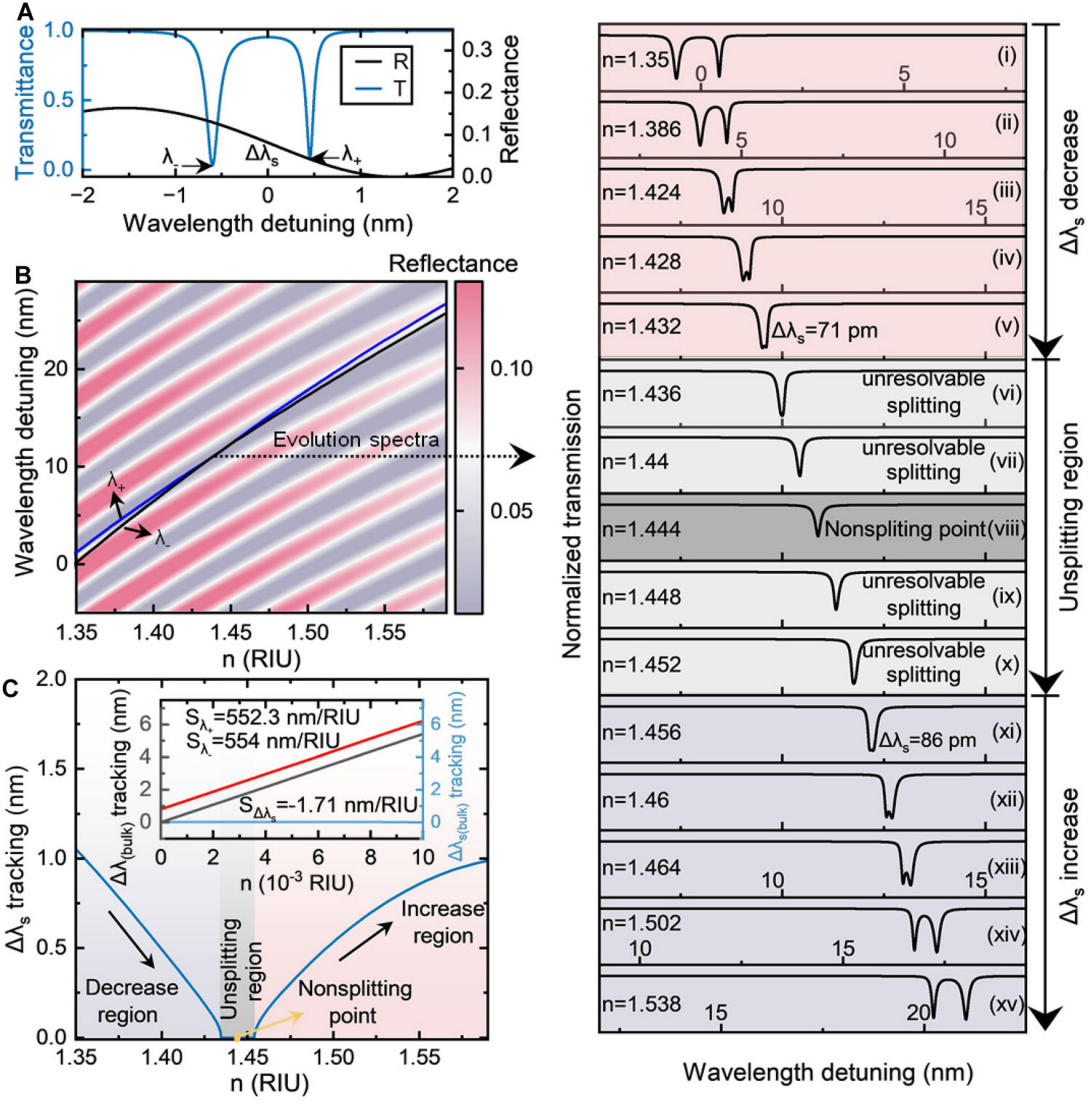
Theoretical evolution of mode splitting with surface index variation. (A) Simulated transmittance and reflectance spectra of the surface‐layer‐covered MWMR at *n* = 1.35. (B) Black (*λ*_−_) and blue (*λ_+_*) curves trace the trajectories of the two resonant wavelengths in (A); right insets show the corresponding spectral evolution. (C) Quantified Δ*λ*_s_ variations with increasing *n*, identifying three regions—decreasing, unsplitting, and increasing—with inset showing the bulk sensitivities of *λ*_−_, *λ_+_*, and Δ*λ*_s_.

Transmission spectra (*T*) of the MWMR, derived by extending this equivalent‐reflection model to the coupled microring, where it serves as an effective phenomenological description of the CW–CCW coupling underlying mode splitting (see Section S4 and Figure S3). Figure 2A shows a typical mode splitting spectrum and the corresponding internal reflection, estimated by the aforementioned meta‐waveguide model. The amount of mode splitting (Δ*λ*_s_), defined as *λ*_+_—*λ*_‐_, is proportional to the strength of the internal reflection [21]. Due to the reflection‐induced wavelength‐dependent loss, the split modes exhibit different quality factors (*Q)* [20]. Figure 2B illustrates the evolution of the internal reflection within the meta‐waveguide induced by variations in the refractive index of the surface layer. The black (*λ*_−_) and blue (*λ*_+_) curves represent the trajectories of the two resonant wavelengths depicted in Figure 2A, respectively. The corresponding spectra are shown in the right insets of Figure 2B. As *n* initially increases, Δ*λ*_s_ decreases, as illustrated in insets (i)–(v). At *n* = 1.436, the splitting becomes indistinguishable due to the limited *Q* factor, as shown in inset (vi), where the reflection intensity is approximately 0.09. With a further increase in *n*, the unresolved resonances in inset (vii) exhibit different *Q* factors and extinction ratios compared to inset (vi), indicating mode broadening in this region [37]. At *n* = 1.444, the reflection drops to zero, and the resonance transitions to an unsplit state, as illustrated in inset (viii). Beyond the zero‐reflection point, unresolvable splitting reappears (insets (ix)–(x)). At *n* = 1.448, mode splitting re‐emerges with a splitting of 71 pm, and its strength continues to grow with increasing *n*, as depicted in insets (xi)–(xv). Figure 2C summarizes the quantified Δ*λ*_s_, identifying three regions: decreasing, unsplitting, and increasing. An extended refractive‐index range is used in the simulation to capture the full evolution of the mode‐splitting behavior, while the experiments probe only a small perturbation within the relevant regime. The unsplitting region includes both the unresolvable splitting and zero‐reflection‐induced nonsplitting states. The decreasing regime is analyzed as a representative case to elucidate the sensing characteristics. Because the splitting response is not strictly linear, the surface sensitivity is expressed as a range of 10–15 nm/RIU. Within the same regime, *λ*_+_ and *λ*_−_ exhibit stronger surface‐induced shifts of 111 and 123 nm/RIU, respectively. To assess practical relevance, the bulk sensitivity is also calculated to evaluate the response to spatially homogeneous refractive‐index variations, such as temperature fluctuations, and other non‐local background changes. As shown in the inset of Figure 2C, variation of the bulk refractive index induces linear shifts of *λ*_+_ and *λ*_−_ (552 and 554 nm nm/RIU), whereas Δ*λ*_s_ remains nearly invariant (−1.7 nm/RIU). These results indicate that, although *λ*_±_ and Δ*λ*_s_ both respond to surface perturbations, Δ*λ*_s_ remains insensitive to bulk variations, establishing it as a stable and reliable sensor for surface‐specific sensing. In contrast, the pronounced bulk sensitivity of *λ*_±_ implies that weak surface signals can be easily masked by background‐index fluctuations, whereas Δ*λ*_s_ effectively isolates the interfacial response and ensures quantitative accuracy even under varying environmental conditions.

### 2.2 Noise‐Resilient Optical Response Enabled by Mode Splitting in MWMRs

**FIGURE 3:**
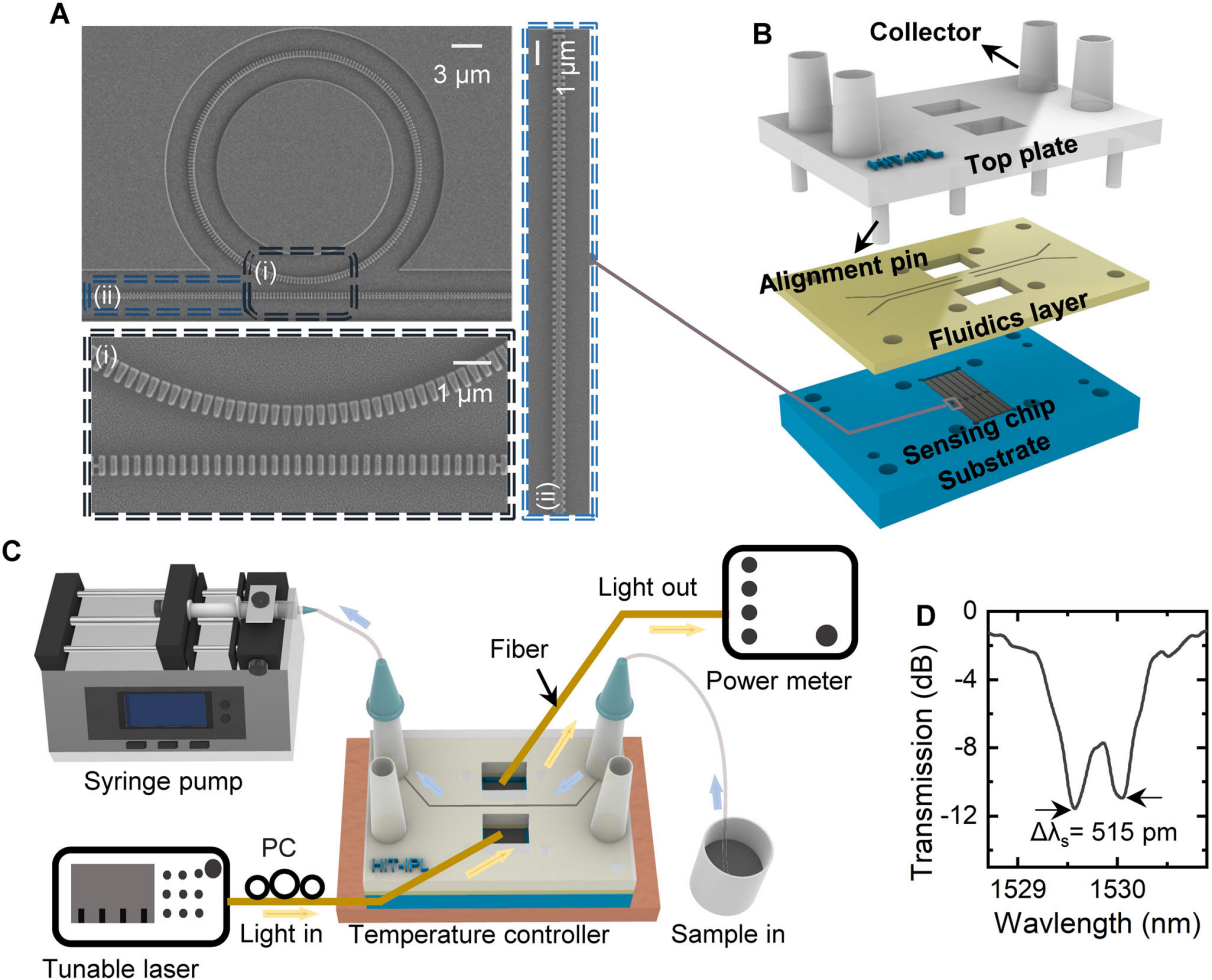
Fabricated MWMR device and experimental setup. (A) SEM images of the fabricated MWMR: (i) coupling region and (ii) adiabatic mode converter (ring radius = 10 µm). (B) Customized microfluidic system. (C) Automated optical testing platform. (D) Transmission spectrum of the MWMR exhibiting mode splitting when immersed in PBS buffer.

To assess the optical performance and stability of the proposed mode splitting‐based MWMR, the fabricated device and experimental setup are shown in Figure 3. Figure 3A shows scanning electron microscopy (SEM) images of the fabricated MWMR. The detailed fabrication process is described in the Materials and Methods section. The insets of Figure 3A illustrate the coupling region (i) and the adiabatic mode converter (ii). The converters are placed at both ends of the meta bus waveguide to facilitate mode conversion between the fundamental mode of the silicon strip waveguide and that of the meta bus waveguide. The fabricated MWMR is encapsulated in a customized microfluidic package and measured with an in‐house automated testing system (see Materials and Methods section, Section S5 and Figure S4), as illustrated in Figure 3B,C. Figure 3D shows the transmission spectrum of the MWMR exhibiting mode splitting when immersed in phosphate‐buffered saline (PBS) buffer. The mode splitting occurs near 1530 nm with Δ*λ*_s_ of 515 pm. This measurement establishes a spectral reference for subsequent sensing analyses.

**FIGURE 4:**
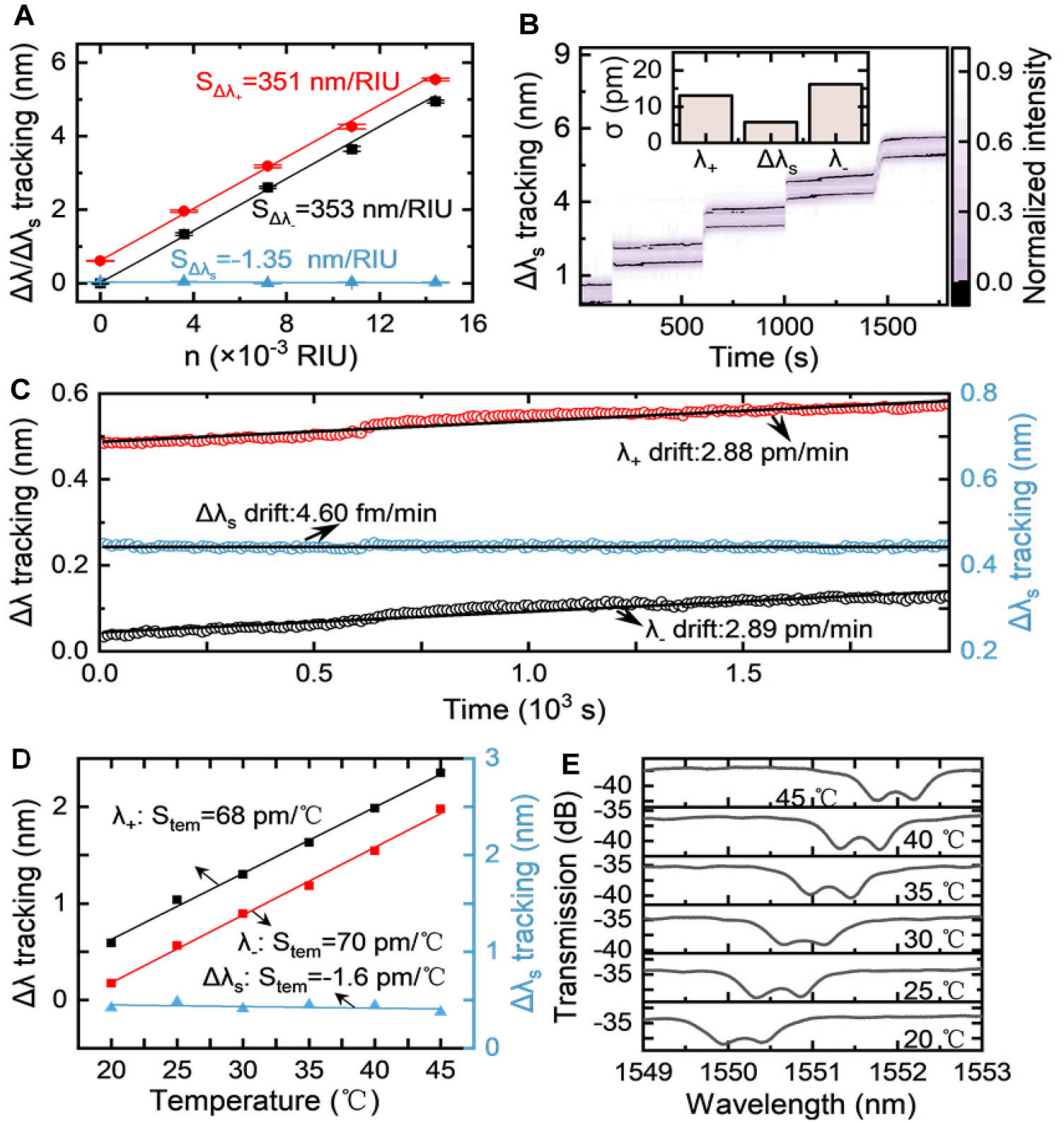
Sensing performance and stability of the mode splitting‐based MWMR. (A) Experimental bulk refractometric sensitivities of *λ_+_*, *λ_−_*, and Δ*λ*_s_. (B) Normalized spectral responses of*λ_+_*, *λ*_−_, and Δ*λ*_s_ under bulk‐index perturbations; inset compares their baseline noise levels (standard deviation). (C) Baseline drift monitoring in PBS after APTES modification. (D) Temperature sensitivities of*λ_+_*, *λ_−_*, and Δ*λ*_s_. (E) Transmission spectra at different stage temperatures.

The bulk refractometric sensitivity (*S*) of the MWMR is quantified using NaCl solutions with defined refractive indices. The resonance shifts are linearly fitted against refractive‐index variations, as summarized in Figure 4A. The fitted slopes are 351 nm/RIU for *λ*_+_, 353 nm/RIU for *λ*_−_, and −1.35 nm/RIU for Δ*λ*_s_, with error bars representing standard deviations (*σ*). Figure 4B shows the normalized spectral responses, and the inset compares the baseline noise of *λ*_+_, *λ*_−_, and Δ*λ*_s_ (13 pm, 16.1 pm, and 5.7 pm, respectively). The LOD, calculated as 3*σ*/*S*, is determined to be 9.82 × 10^−^^5^ RIU (details in Section S6). These results agree well with the simulation, confirming that Δ*λ*_s_ is insensitive to homogeneous bulk‐index variations and thus exhibits intrinsic immunity to such perturbations.

The temporal stability of the MWMR is evaluated by analyzing its baseline drift, which is a deterministic factor for achieving an ultra‐low LOD [38]. Baseline drift must be kept small enough compared with the biomolecule binding signals. This drift can result from factors such as temperature differences between two solutions and surface oxidation, which contribute to non‐negligible signal interferences [36]. Therefore, precise and costly environmental controls are often necessary for conventional sensors to stabilize the sensing system. To demonstrate the effectiveness of the proposed self‐referenced sensor, the spectral responses are continuously monitored for 2000 s while the device is immersed in PBS buffer after the 3‐aminopropyl triethoxysilane (APTES) monolayer modification (see Materials and Methods), as shown in Figure 4C. Both *λ*_+_ and *λ*_−_exhibit significant red‐drift originating from the aforementioned factors at comparable rates (2.88 pm/min and 2.89 pm/min, respectively; statistical analysis can be found in Section S7), whereas Δ*λ*_s_ remains stable with a drift of merely 4.6 fm/min, which is three orders of magnitude lower than the wavelength‐shift based interrogation method. This performance is also comparable to that of commercial instruments, such as the Biacore 1 series model, which employs a microfluidic parallel reference channel to suppress blank drift to less than 0.003 RU/min (= 3 fm/min) [39]. The temperature sensitivity of the mode splitting‐based MWMR is evaluated under varying environmental conditions, as shown in Figure 4D,E. The high thermo‐optic coefficient of silicon (1.86 × 10^−^^4^/K) [40] makes silicon‐based devices intrinsically sensitive to thermal perturbations. Accordingly, both resonances of the mode splitting doublet are red‐shifted as the temperature increases from 20°C to 45°C, with fitted temperature sensitivities of 68 pm/°C for *λ*_+_ and 70 pm/°C for *λ*_−_. In contrast, the Δ*λ*_s_ changes by only −1.6 pm/°C—approximately 43‐fold smaller than the individual shifts. This pronounced disparity indicates that mode splitting readout effectively suppresses temperature‐induced fluctuations, ensuring stable sensor operation under practical conditions. Overall, these analyses substantiate Δ*λ*_s_ as an intrinsically bulk‐insensitive and environmentally robust spectral indicator, underscoring its potential for high‐stability surface‐sensing applications.

### 2.3 Detection of Ultra‐Weak Surface Perturbation

**FIGURE 5:**
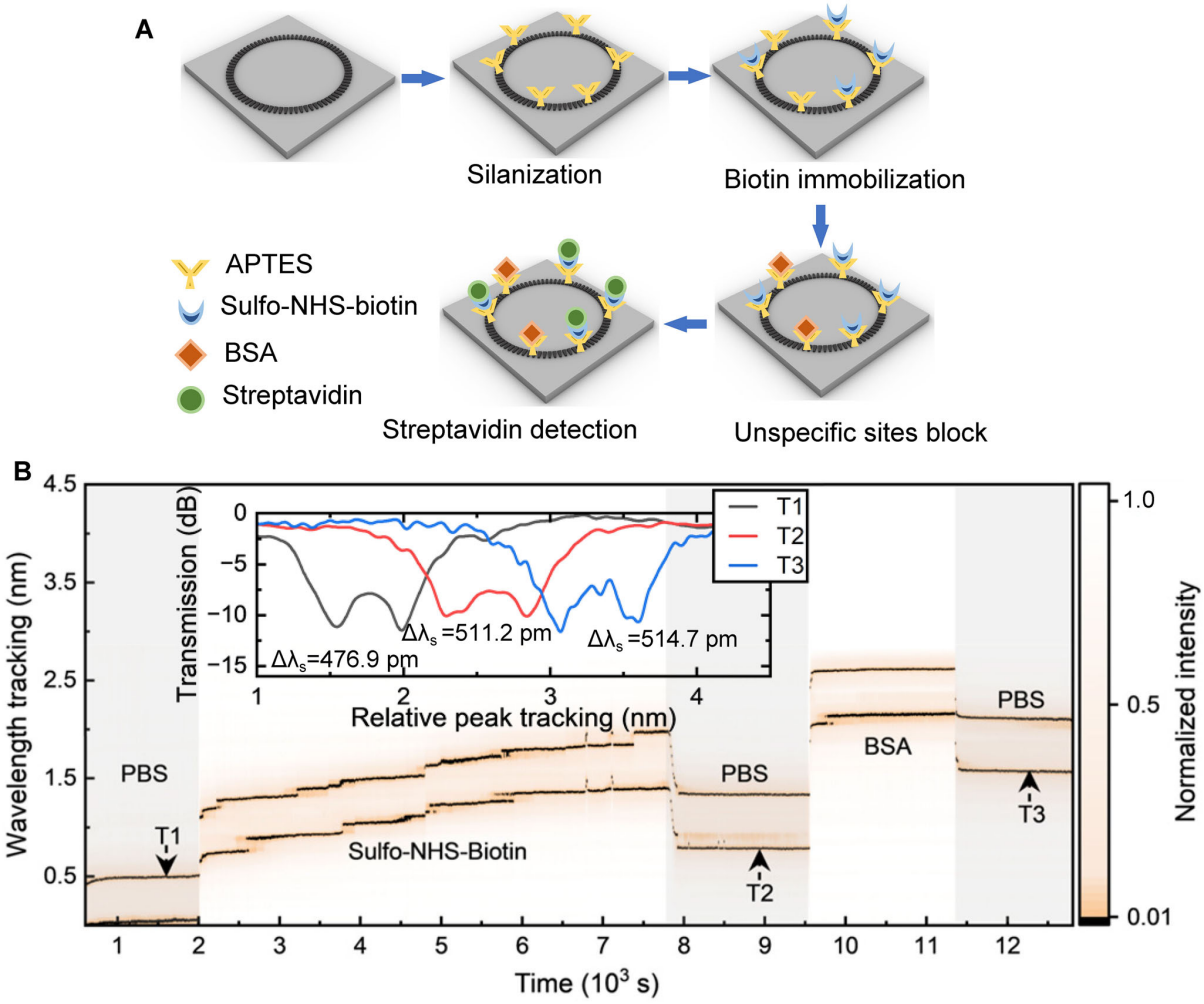
Surface functionalization responses of the mode splitting‐based MWMR. (A) Schematic illustration of surface functionalization, including Sulfo‐NHS‐Biotin immobilization, BSA blocking, and subsequent streptavidin detection. (B) Normalized spectral responses of the splitting modes during Sulfo‐NHS‐Biotin immobilization and BSA blocking. Insets show mode splitting spectra at equilibrium states T1 (baseline), T2 (after Sulfo‐NHS‐Biotin immobilization followed by PBS washing), and T3 (after BSA blocking followed by PBS washing).

The ability of mode splitting‐based MWMRs to resolve interfacial ultra‐weak perturbations is validated using the streptavidin–biotin interaction as a representative model system. In this experiment, the binding process provides a controlled and quantifiable refractive index variation at the waveguide surface, enabling systematic assessment of the platform's detection capability. The surface functionalization procedure, illustrated in Figure 5A, involved immobilizing 10 mg/mL Sulfo‐NHS‐Biotin onto the APTES layer, followed by blocking with 1% BSA to prevent non‐specific binding (details in the Materials and Methods). High‐concentration probe and blocking solutions are used to maximize the probe coverage of the sensor surface. The signals harvested during the initial PBS wash serve as the baseline for subsequent measurements. Figure 5B summarizes the spectral responses throughout the entire surface functionalization procedure. Spectra at equilibrium states T1 (baseline), T2 (after Sulfo‐NHS‐Biotin immobilization followed by PBS washing), and T3 (after BSA blocking followed by PBS washing) illustrate the evolution of mode splitting, as shown in the inset of Figure 5B. Δ*λ*_s_ at these states are 476.9 pm, 511.2 pm, and 514.7 pm, respectively.

**FIGURE 6:**
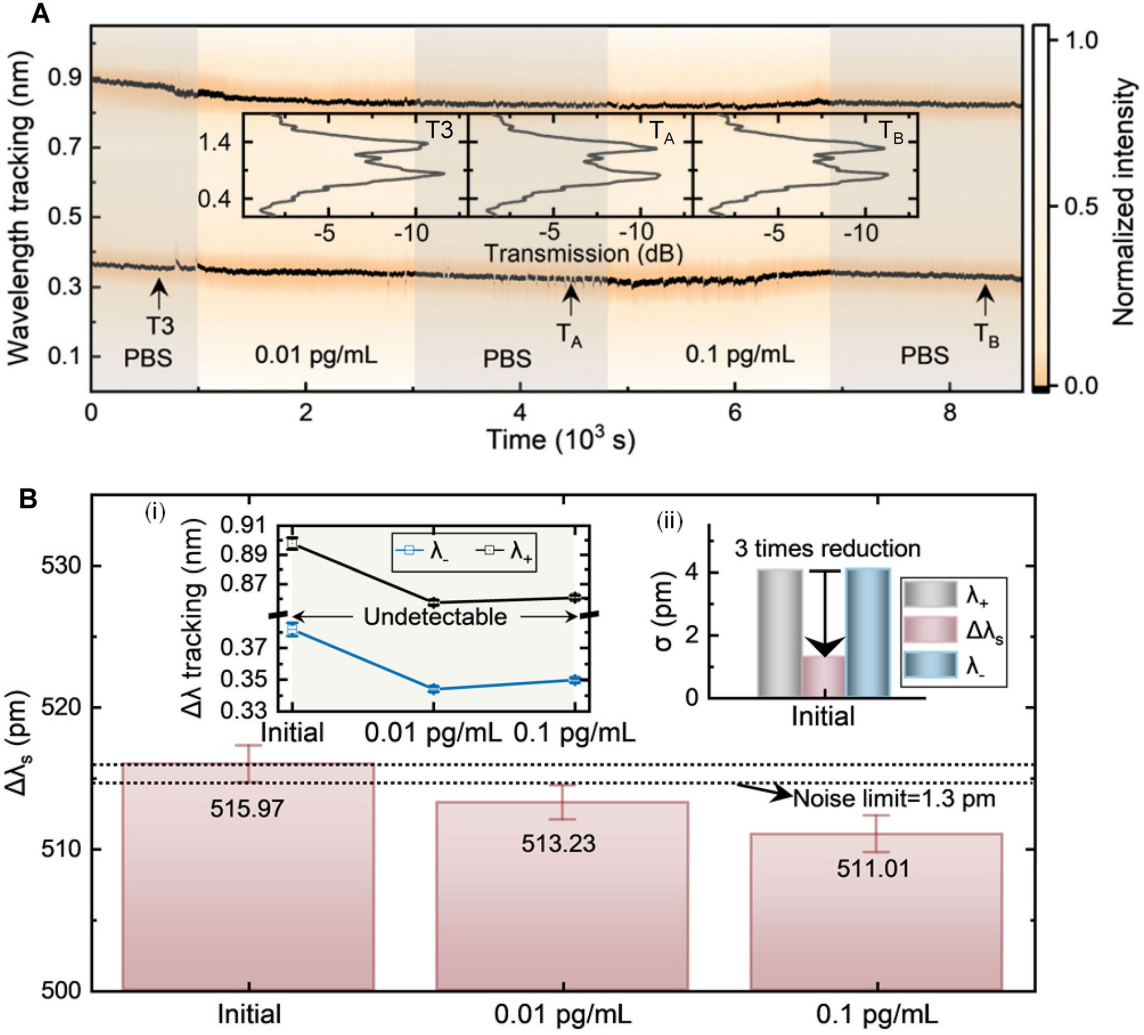
Ultra‐low‐concentration streptavidin detection. (A) Temporal trace of Δ*λ*_s_ extracted from sequential measurements of streptavidin at different concentrations with PBS washing. Insets show equilibrium mode splitting spectra at T3 (after BSA blocking and PBS washing), T_A_ (after 0.01 pg/mL streptavidin injection and PBS washing), and T_B_ (after 0.01 pg/mL streptavidin injection and PBS washing). (B) Quantified responses of *λ*_+_, *λ*_−_, and Δ*λ*_s_ at different streptavidin concentrations. Insets: (i) individual resonance blue shifts of *λ*_+_ and *λ*_−_; (ii) baseline noise comparison among *λ*_+_, *λ*_−_, and Δ*λ*_s_.

Following functionalization, various concentrations of streptavidin solutions are measured sequentially, with PBS flowing after each step to remove unbound streptavidin molecules. Figure 6A shows the temporal trace of Δ*λ*_s_ extracted from over 850 spectra. The equilibrium spectra at T3 (after BSA blocking and PBS washing), T_A_ (after the injection of 0.01 pg/mL streptavidin followed by PBS washing), and T_B_ (after the injection of 0.1 pg/mL streptavidin followed by PBS washing) illustrate the evolution of mode splitting, as shown in the insets of Figure 6A (additional concentration responses can be found in Section S8 and Figure S5). The corresponding time‐dependent mode splitting Δ*λ*_s_(t) at different equilibrium states is provided in Section S9 and Figure S6). Using the averaged initial mode splitting value of 515.97 pm as the baseline, Figure 6B summarizes the quantified responses of *λ*_+_, *λ*_−_, and Δ*λ*_s_ to two ultra‐low concentrations of streptavidin. Blue shifts of individual resonance (*λ*_+_ or *λ*_−_) are observed at both concentrations, indicating that the detectable range for the wavelength shift‐based interrogation method exceeds 0.1 pg/mL, as shown in inset (i) of Figure 6B. The observed blue shifts in *λ*_+_ and *λ*_−_ are likely attributed to probe detachments and/or silicon etching in biological solutions, as suggested by recent studies [39]. In contrast to resonance wavelength shifts, fluctuations in mode splitting are considerably weaker during measurement, since mode splitting depends on changes in the reflectivity of the meta‐grating induced by the accumulation of biomolecules rather than variations in the effective index of the modes within the ring [24]. As shown in inset (ii) of Figure 6B, the *σ* of Δ*λ*_s_ (1.3 pm) is reduced by more than threefold compared to the individual wavelength deviations of *λ*_+_ (4.1 pm) and *λ*_−_ (4.07 pm). Δ*λ*_s_ decreases by 2.74 pm upon applying 0.01 pg/mL streptavidin, indicating that the MWMR can detect streptavidin at this ultra‐low concentration, as shown in Figure 6B. For 0.1 pg/mL streptavidin, Δ*λ*_s_ further decreased by 2.22 pm. Compared with the detectable concentration achieved by the wavelength‐shift approach (higher than 0.1 pg/mL), the mode splitting scheme reduces the detectable concentration by at least two orders of magnitude. This improvement arises from the discernible yet stable mode splitting response to interfacial refractive‐index perturbations, whereas its negligible sensitivity to homogeneous background variations ensures reliable identification of surface‐binding events. It should be noted that the mode splitting‐based interrogation is intrinsically nonlinear, with concentration‐dependent sensitivity governed by the underlying mode‐coupling dynamics. As a result, the response is not intended to be strictly linear over a broad range, but instead optimized for detecting subtle perturbations in the ultra‐low‐concentration regime relevant to lethal pathogens (see Section S10 and Figure S7).

## 3 Discussion

This study demonstrates that harnessing residual reflection in MWMRs enables mode splitting to serve as a self‐referenced architecture for surface refractive‐index sensing. Unlike conventional wavelength‐shift interrogation, which is highly susceptible to baseline drift and environmental fluctuations and often fails to resolve ultra‐weak surface perturbations, mode splitting exhibits intrinsic stability and strong immunity to noise owing to its differential nature. Experimentally, mode splitting shows an exceptionally low baseline drift of only 4.6 fm/min. Its modest temperature sensitivity (−1.6 pm/°C) further confirms robust operation without stringent temperature control. Interfacial refractive‐index perturbations modulate the reflection and consequently alter Δ*λ*_s_, allowing quantitative yet noise‐resilient interrogation of surface processes. The streptavidin–biotin assay corroborates this mechanism, revealing discernible splitting variations upon molecular binding and confirming a detection limit of 0.01 pg/mL with a splitting noise of 1.3 pm. These results validate that reflection‐induced mode splitting effectively decouples surface‐specific responses from bulk and environmental disturbances, enabling reliable identification of ultra‐weak interfacial events.

Table 1 summarizes surface sensing performance reported for various microring‐based structures. Wavelength‐shift‐based strip microrings generally exhibit detection limits within the pg/mL to ng/mL range (Refs [6, 7]), while pedestal‐type MWMRs further improve structural sensitivity, reaching 0.1 ng/mL [17]. Compared to sensors based on wavelength shift, mode splitting‐based MWMRs achieve 2–5 orders of magnitude improvement in detectable concentrations. Additionally, compared with other mode‐splitting‐based sensors such as Bragg‐grating microrings for rhS100A4 [24] and EP‐based sensors for single‐particle detection [42], the mode splitting‐based MWMR achieves substantially improved detection performance while maintaining strong noise immunity. As noted in Ref.  [38], achieving a low LOD in biosensing requires minimizing both baseline drift and noise, with Biacore defining LOD as (baseline drift+3 × noise standard deviation). The mode splitting‐based MWMR intrinsically satisfies this requirement through its self‐referenced optical splitting mechanism, effectively reducing both baseline drift and bioprocess‐induced fluctuations.

**TABLE 1:** The sensing performance of microring‐based sensors.

Structure	Method	Analyte	Achieved detectable concentration	Ref
Cascaded microring	Wavelength shift	HSA	18.09 ng/mL	[6]
Strip microring	Wavelength shift	Streptavidin	∼60 fM^a^ (estimate value∼3.59 pg/mL)	[7]
MWMR	Wavelength shift	Streptavidin	10 µg/mL	[43]
Pedestal‐MWMR	Wavelength shift	Streptavidin	0.1 ng/mL	[17]
Bragg‐gating‐based microring	Mode splitting	rhS100A4	1 µM	[24]
EP‐based microring	Mode splitting	Polystyrene nanoparticles	20 fM^b^	[42]
This work (mode splitting‐based MWMRs)	Mode splitting	Streptavidin	0.01 pg/mL (∼0.167 fM)	

^a^ The strip microring with an estimated detectable concentration of 3.59 pg/mL, attributed to its superior relative spectral resolution and an extremely low noise limit of 0.31 pm (3σ), achieved through simultaneous sweeping with a 25‐GHz Fabry‐Perot etalon.

^b^ The EP‐based microring is used for detecting polystyrene (PS) particles, with the concentration recorded here referring specifically to the concentration of PS particle solutions.

These attributes make the mode‐splitting‐based MWMR architecture particularly advantageous for sensing scenarios demanding simultaneous high sensitivity and noise robustness. Beyond the demonstrated streptavidin–biotin assay, this platform could be readily extended to nanoscale biological targets such as extracellular vesicles, which play pivotal roles in immune regulation, coagulation, and intercellular communication [45, 46, 47]. The broader extensibility of this platform is also supported by recent demonstrations of meta‐ring resonators on the SiN platform [48]. Collectively, these results establish reflection‐induced mode splitting as a powerful photonic mechanism that enables drift‐immune, trace‐level detection, positioning MWMRs as a high‐performance and broadly applicable platform for precision surface refractometric sensing.

## 4 Materials and Methods

### 4.1 Fabrication of the MWMR

The structures are fabricated on a silicon‐on‐insulator (SOI) wafer, consisting of a 220‐nm‐thick single‐crystal silicon layer separated from the silicon handle wafer by a 2‐µm‐thick buried oxide (SiO_2_) layer. The diluted ZEP520A resist is spin‐coated on a Piranha‐cleaned chip and baked at 180°C for 20 min before E‐beam lithography (NanoBeam, nB5) to ensure successful nanostructure fabrication. The designs are then transferred to the silicon layer using inductively coupled plasma (ICP) etching. A 10‐nm‐thick silica layer is deposited on the MWMRs via plasma‐enhanced chemical vapor deposition (PECVD) to protect the device from bioassays and enhance salinization performance.

### 4.2 Surface Functionalization and Sample Preparations

A self‐assembled monolayer of 3‐Aminopropyl triethoxysilane (APTES) is formed on the surface of the MWMR, providing amine groups (─NH_2_) for binding probe proteins. The chip is baked at 120°C for 1 h under ambient conditions to enhance the stability of the functional groups. After APTES modification, 10 mg/mL Sulfo‐NHS‐Biotin (Sigma‐Aldrich), a receptor of streptavidin, is flowed to the APTES/MWMR through the microfluidic channel by syringe pump with a pumping speed of 50 µL/min for 1 h. After immobilizing the probes, the remaining chip surface is blocked with 1% BSA in PBS to minimize non‐specific binding. The standard streptavidin (SA) powder (Sigma‐Aldrich) is prepared into 0.01 pg/mL, 0.1 pg/mL, 1 pg/mL, 10 pg/mL, and 100 pg/mL in a solution of PBS for studying the sensitivity of the mode splitting‐based MWMRs.

### 4.3 Microfluidic System

The microfluidic system is illustrated in Figure 3B, which employs a sandwich structure for encapsulation, composed of a top plate, a fluidics layer, and a substrate plate with a rectangular pocket for holding a chip in place. The sample sources are injected into the microfluidic channel from the inflow tube. A syringe for pumping the biomolecules is connected to the outflow tube by a stepping motor to control the flow rate. The standard Luer taper is integrated with the collector for fluid flow in the circuit.

### 4.4 Automatic Testing System

Light from a tunable laser (Santec TSL550), controlled by a polarization controller, is coupled into and out of the chip using grating couplers (more details can be found in Section S11 and Figure S8). The stage temperature is controlled at 25°C with an Apico (AP‐TEC‐PRO) temperature controller to avoid thermal effect‐induced resonance shift during biosensing testing. The transmission spectra are monitored by the optical power meter (Santec MPM‐210H).

## Conflicts of Interest

The authors declare no conflict of interest.

## Supporting information




**Supporting File**: advs75339‐sup‐0001‐SuppMat.docx.

## Data Availability

The data that support the findings of this study are available on request from the corresponding author.
