# Supplementary material for: Breaking the Bottleneck in Limit of Detection of Surface Refractive Index Sensing by Harnessing Meta‐Waveguide Microring Resonators
Source: Adv Sci (Weinh). 2026 Apr 21;13(38):e75339. doi: 10.1002/advs.75339 (PMC13335428; doi:10.1002/advs.75339)
Supplement: Supplementary file 1 — Supporting File: advs75339‐sup‐0001‐SuppMat.docx. [file ADVS-13-e75339-s001.docx]

Supplementary Materials

**Breaking the Bottleneck in Limit of Detection of Surface Refractive Index Sensing by Harnessing Meta-Waveguide Microring Resonators**

*Wanxin Li, Jiewen Li, Rui Li, Gina Jinna Chen, Perry Ping Shum, Yi Zou*, Ray T. Chen, Yongkang Dong*, and Xiaochuan Xu**

W. Li, C, J. Li, R. Li, Y. Dong, X. Xu

National Key Laboratory of Laser Spatial Information, Guangdong Provincial Key Laboratory of Integrated Photonic-Electronic Chip, Guangdong Provincial Key Laboratory of Aerospace Communication and Networking Technology, Harbin Institute of Technology, Xili University Town, Harbin Institute of Technology campus, Shenzhen, Guangdong 518055, China

E-mail: aldendong@163.com; xuxiaochuan@hit.edu.cn

G. J. Chen, P. P. Shum

State Key Laboratory of Optical Fiber and Cable Manufacture Technology, Guangdong Key Laboratory of Integrated Optoelectronics Intellisense, Department of EEE, Southern University of Science and Technology, Shenzhen 518055, China

Y. Zou

School of Information Science and Technology, ShanghaiTech University, Shanghai 201210, China

E-mail:zouyi@shanghaitech.edu.cn

R. T. Chen

Department of Electrical and Computer Engineering, The University of Texas at Austin, 10100 Burnet Rd., Austin, Texas 78758, USA

S1 Advantages of mode splitting sensing in meta-waveguide microrings

Mode splitting typically arises from mutual coupling between clockwise (CW) and counterclockwise (CCW) modes in whispering gallery mode (WGM) resonators, induced by reflective elements, such as sidewall roughness^[1]^, scatters^[2–4]^, and periodic structures^[5–10]^, which lift mode degeneracy. However, not all reflective elements improve sensing performance. For instance, sidewall roughness, which exists in any ring, is random and can severely degrade the quality (*Q*) factor and weaken photon-matter interaction. Scatters are often added as additional elements to modulate microring reflections, but the system remains limited by the evanescent field of conventional waveguides. Microrings with periodical structures provide a deterministic reflection channel to generate and control mode splitting properties, such as resonance wavelength and splitting magnitude^[5, 9]^, through sinusoidal structures^[8]^, Bragg gratings^[6]^, and meta-waveguides^[5]^. While optimizing the periodic modulation within microrings for mode splitting sensing, it is essential to consider the tradeoff between the quality (*Q*) factor and the overlap of the enhanced fields with target analytes. Among these periodical structures, meta-waveguide microring resonators (MWMRs) are particularly advantageous because they harness residual reflection to sustain lossless modes with significant field overlap in the sensing medium.

S2 Reflection in meta-structure

For an ideal infinitely periodic subwavelength meta-waveguide, the propagating Bloch mode is an eigenstate of the periodic system and therefore does not exhibit reflection during propagation. In the present work, however, we are concerned with practical finite structures rather than an ideal infinite Bloch system. In the straight-waveguide case, this means that a finite-length meta-section is excited through mode conversion from a conventional strip-waveguide mode. In the microring case, the periodic section is likewise finite, while the resonator is additionally lossy and has a finite *Q* factor, corresponding to a finite photon lifetime and effective interaction length. Accordingly, our focus here is on the weak reflection-/back-coupling-related response that arises in such practical finite structures.

As shown in Figure S1(A), the operating point of the 300 nm grating used in this work remains in the subwavelength regime and outside the Bragg bandgap, while already lying in a regime where reflection-related spectral response in the finite meta-waveguide becomes observable. To quantify this behavior, finite-difference time-domain (FDTD) simulations were performed for finite-length meta-waveguides with grating periods of 200 nm and 300 nm while keeping the duty cycle (*DC*) identical. The resulting transmission spectra are shown in Figure S1(B). Compared with the 200 nm case, the 300 nm grating exhibits a more pronounced reflection-related spectral response within the C+L band. This indicates that, for the finite-length periodic structures considered here, the reflection-related response in the target wavelength range becomes stronger as the operating condition moves closer to the Bragg-related regime. Consequently, for the grating parameters employed in this work, the reflection-/back-coupling-related response associated with the finite-length periodic section cannot be neglected within the operating wavelength range.

| 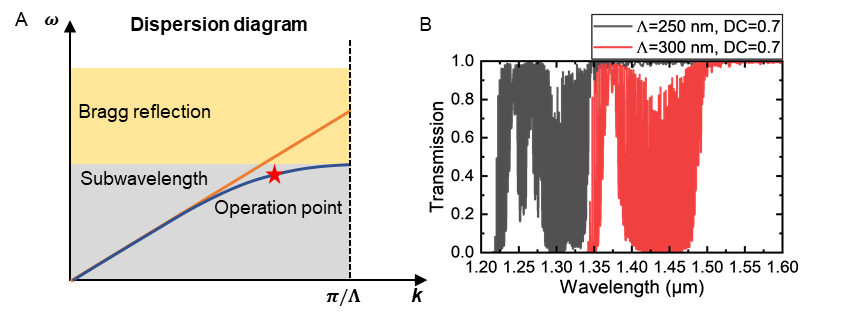 |
| --- |
| Figure S1. (A) Schematic dispersion diagram of a periodic structure. (B) Simulated transmission spectra of meta-waveguides with grating periods of 250 nm and 300 nm under identical duty cycles. |

S3 Transfer matrix of the multilayer model

The reflection characteristics of the meta-waveguide are modeled as a multilayer stack consisting of silicon (material 1), cladding (material 2), and a surface layer (material 3), as shown in Figure S2. For light propagates in segment *j* (where *j*=1, 2, 3), the phase change is given by *φ*_j_ =(*n*_effj_k-i*α*/2)*d*_j_, where k is the wavenumber in vacuum, *n*_effj_ is the effective refractive index, i is the imaginary unit, *d*_j_ is the medium length and *α* represents the propagation loss, respectively. The transfer matrix$\Phi$can be written as: ^[11–13]^

The interface matrix *S* describes the transmittance and reflectance at the boundaries among three segments, with its elements *r* and *t* derived from the Fresnel formulas for reflection and transmission, respectively. The subscripts of *S* represent the materials’ interfaces and the direction of propagation. *S* can be written as:^[11–13]^

| 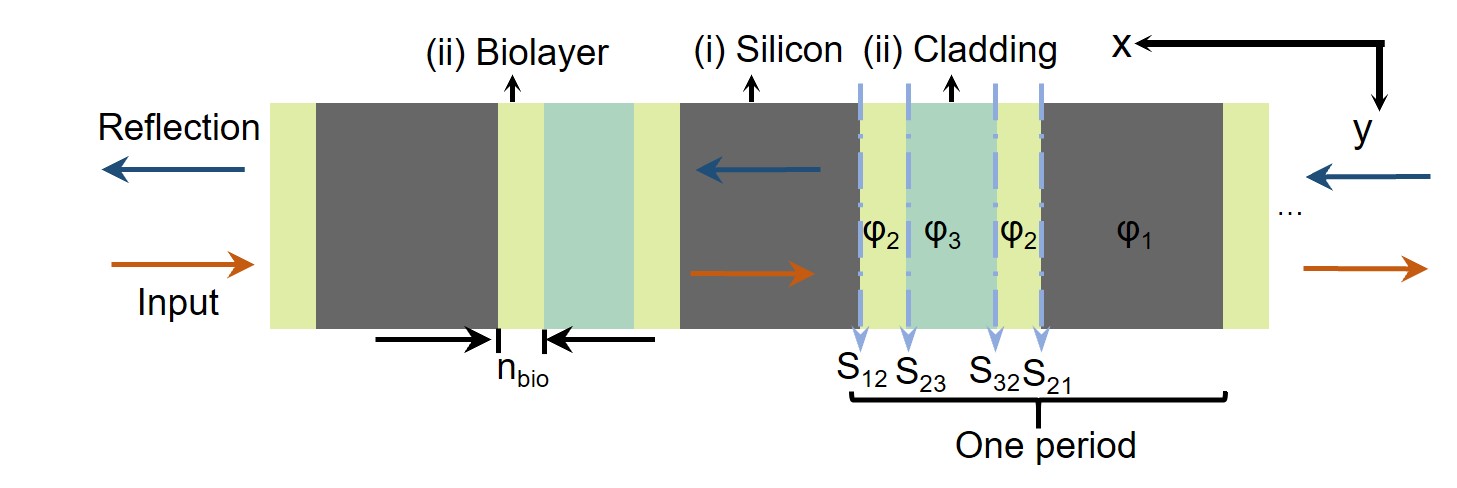 |
| --- |
| Figure S2. The multilayer stack model of the meta-waveguide covered with the surface layer. |

$$S_{mn}=\left(\begin{matrix} \frac{1}{t} & \frac{r}{t} \\ \frac{r}{t} & \frac{1}{t} \end{matrix}\right),m,n\in\left\{1,2,3\right\},m\neq n$$

The wave-transfer matrix *M*_0_ for the one period of the meta-waveguide is obtained by simply multiplying the S/Φ matrices for the individual elements: ^[11–13]^

$$M_{0}=S_{12}\Phi_{2}S_{23}\Phi_{3}S_{32}\Phi_{2}S_{21}\Phi_{1}$$

The wave-transfer matrix of N periods of the meta-waveguide can be expressed as**. Then, the reflection *R* of the meta-waveguide can be readily calculated by$R=\left|M_{21}/M_{22}\right|^{2}$.^[12, 13]^

S4 Transfer matrix of the modified microring model

| 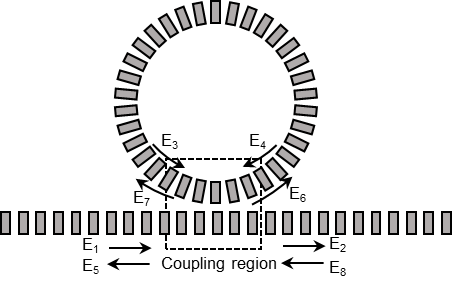 |
| --- |
| Figure S3. The model of MWMR coupled to a meta-waveguide. |

The transmission characteristics of the MWMR can be analyzed by adapting the simplified model from Refs.[5,12,13], where the reflection of the MWMR is treated as equivalent to a reflector in a microring resonator. The phase discrepancy (*φ_c_*) between the optical mode and the periodic modulation introduced by the meta-waveguide’s large mode field also affects the transmission spectrum features of the MWMR. Thus, the transfer matrix is modified as. Following simplified model, the field amplitudes at the point of the waveguide–resonator coupling are defined in Figure S3 for possible waves^[12]^. *E*_1_​ and *E*_2​_ denote the incident and transmitted fields in the bus waveguide, respectively, *E*_5_​ denotes the reflected field, and *E*_3_​, *E*_4_​, *E*_6_, and *E*_7_ denote the counter-propagating circulating fields at the coupling point. Here, *E*_8_=0 corresponds to the single-port input condition. The fields inside the finite periodic section satisfy:

$$\left[\begin{matrix} E_{3} \\ E_{7} \end{matrix}\right]=\left[\begin{matrix} M_{11} & M_{12} \\ M_{21} & M_{22} \end{matrix}\right]\left[\begin{matrix} E_{4} \\ E_{6} \end{matrix}\right]$$

At the bus waveguide-ring coupling region, the field relations are given by:

$$\left[\begin{matrix} E_{5} \\ E_{7} \end{matrix}\right]=\left[\begin{matrix} -it & \kappa\\ \kappa& -it \end{matrix}\right]\left[\begin{matrix} E_{8} \\ E_{6} \end{matrix}\right]$$

$$\left[\begin{matrix} E_{4} \\ E_{2} \end{matrix}\right]=\left[\begin{matrix} -it & \kappa\\ \kappa& -it \end{matrix}\right]\left[\begin{matrix} E_{1} \\ E_{3} \end{matrix}\right]$$

where$\kappa$and$t$are the reflection and transmission coefficients, respectively, and satisfy$\kappa^{2}+t^{2}=1$. By combining Eqs. (4)–(6) with the coupling relations, the transmission of the device can be obtained as**:**

$$\left|T\right|=\left|\frac{E_{2}}{E_{1}}\right|=\left|\frac{-1-\kappa^{2}+\kappa(M_{11}+M_{22})}{M_{22}-2\kappa+\kappa^{2}M_{11}}\right|$$

In this formulation, the total finite meta-waveguide section is represented as an equivalent internal scattering element, whose response is linked to the overall transfer characteristics of the microring. Therefore, the observed mode splitting is described as arising from effective CW-CCW coupling induced by the finite periodic section, determined by the reflection.

S5 The microfluidic system and the automatic test system.

Figure S4 illustrates the microfluidic and automated testing systems. Figure S4(A) shows the packaged microfluidic box containing the sensing chip. The microfluidic device comprises a top plate, a fluidics layer, and a substrate with a rectangular pocket for positioning the chip, as depicted in Figure S4(B). The sample sources are injected into the microfluidic channel from the inflow tube. A syringe for pumping the analytes is connected to the outflow tube by a stepping motor to control the flow rate. The standard Luer taper is integrated with the collector for fluid flow in the circuit. Figure S4(C) shows the automated testing system. Light from a tunable laser (Santec TSL550), controlled by a polarization controller, is coupled into and out of the chip using grating couplers.^[15]^ The stage temperature is controlled at 25 ℃ with an Apico (AP-TEC-PRO) temperature controller to avoid thermal effect- induced resonance shift during sensing testing. The transmission spectra are monitored by the optical power meter (Santec MPM-210H).

| 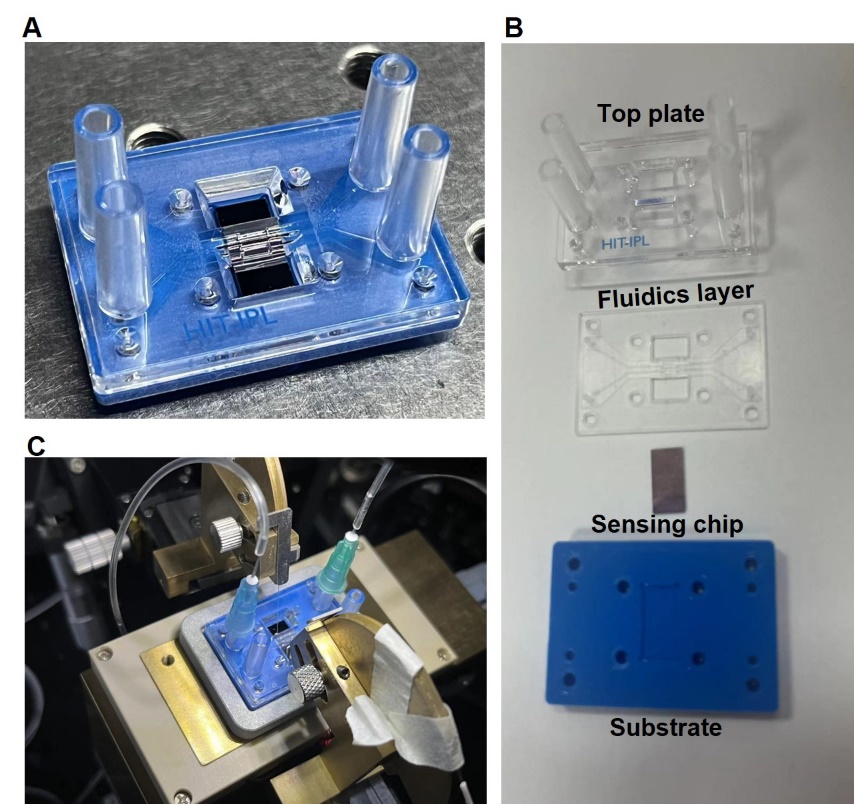 |  |
| --- | --- |
| Figure S4. Microfluidic system and automated testing setup. (A) Packaged microfluidic box. (B) Components of the microfluidic box. (C) Automated testing system. |  |
| S6 Bulk RI Sensitivity and Split‑Assisted LOD  Bulk refractometric sensitivity is calibrated using five NaCl solutions (2% concentration increment corresponding to Δn = 0.0036). For each concentration step, resonance wavelengths are grouped, and the mean and standard deviation are calculated. The averaged split resonances *λ*_+_ and *λ*_−_ are processed as: *avg* = (*λ*_+_+*λ*_−_)/2 and Δ*λ*_s_ =*λ*_+_-*λ*_−_. To suppress correlated drift noise, a split-assisted readout is constructed as: $y=avg-\beta\cdot\Delta\lambda_{s}, \beta=Cov(avg,\Delta\lambda_{s})/Var(\Delta\lambda_{s})$   where *β* was estimated from a baseline segment. For each readout$z\in\{\lambda_{+},\lambda_{-},\text{ }avg,\text{ }y\}$, the group-averaged resonance shift is fitted by: $z\left(\Delta n\right)\text{ }=\text{ }a_{z}+\text{ }S_{z}\cdot\Delta n$   Baseline fluctuations are quantified as σ_z_ (standard deviation), and the LOD is obtained as: $LOD_{z}=3\sigma_{z}/\left\vert\text{ }S_{z}\right\vert$   Using the split-assisted readout, the baseline noise is reduced, giving LOD_y_ = 9.82×10⁻⁵ RIU. |  |
| S7 The Statistical analysis of mode splitting responses  To provide a more rigorous evaluation, the baseline drift, along with its standard deviation and 95% confidence intervals, is summarized in Table 1:  Table 1. Statistical analysis of mode splitting responses   \|  \| Baseline drift \| Standard deviation \| 95% Confidence intervals \| \| --- \| --- \| --- \| --- \| \| *λ*_+_ \| 2.89 pm/min \| 69.53 fm/min \| (2.76, 3.03) pm/min \| \| *λ*_-_ \| 2.88 pm/min \| 64.59 fm/min \| (2.76, 3.02) pm/min \| \| Δ*λ*_s_ \| 4.60 fm/min \| 25.21 fm/min \| (-44.87, 53.97) fm/min \| |  |
| S8 The spectral responses for detecting different concentrations of streptavidin   \| 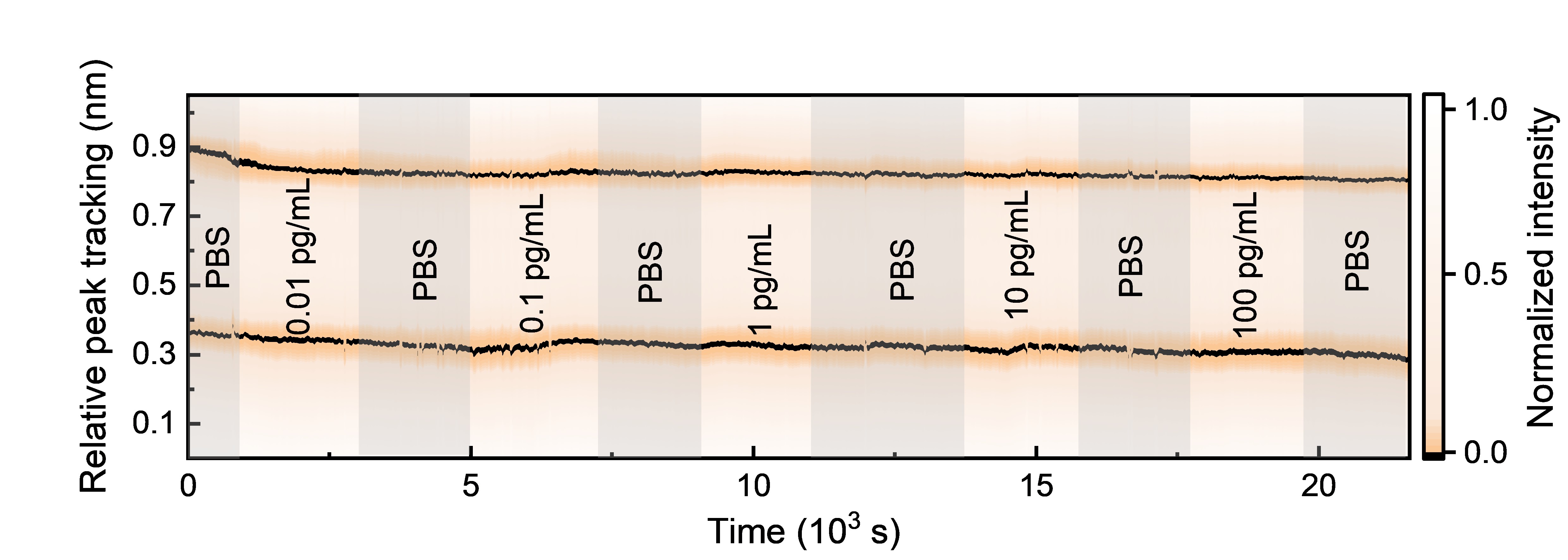 \|  \| \| --- \| --- \| \| Figure S5. The spectral responses of splitting modes to different concentrations of streptavidin with normalized intensity. \|  \| | |

S9 Mode splitting measured under equilibrium periods.

| 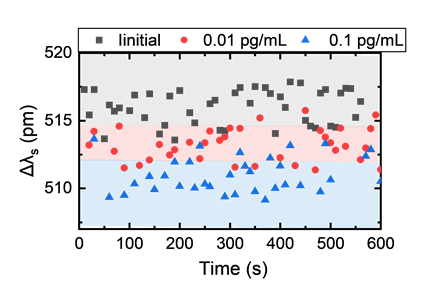 |
| --- |
| Figure S6. The corresponding time-dependent mode splitting Δλ_s_(t) at different equilibrium states. |
|  |

S10 The nonlinear variations of mode splitting

The mode splitting interrogation method exhibits poor linearity, indicating that sensitivity is influenced by changes in the refractive index of the surface layer. This nonlinearity results from the wavelength-dependent nature of reflection, as illustrated in Figure 1(C). Additionally, the reflection intensity changes due to the decreasing refractive index contrast between materials as biomolecules accumulate, further contributing to the nonlinear variations in mode splitting responses. Beyond these factors, the nonlinearity is also affected by the wavelength-dependent coupling between the bus meta-waveguide and the MWMR. Figure S7(A) compares the effects of constant and wavelength-dependent coupling coefficients (*κ*) on mode splitting variations as the refractive index of the surface layer changes, with the inset showing the curves for both coupling coefficients. The Δ*λ*_s_ exhibits better linearity with wavelength-dependent****than with constant *κ* (*κ*=0.9). Figure S7(B) presents five spectra at representative points (A, B, C, D, and E) to illustrate the evolution of mode splitting, with dashed and solid lines representing constant and wavelength-dependent *κ*, respectively. Considering all these factors, the nonlinearity in mode splitting makes the sensor more suitable for detecting target molecules at ultra-low concentrations.

| 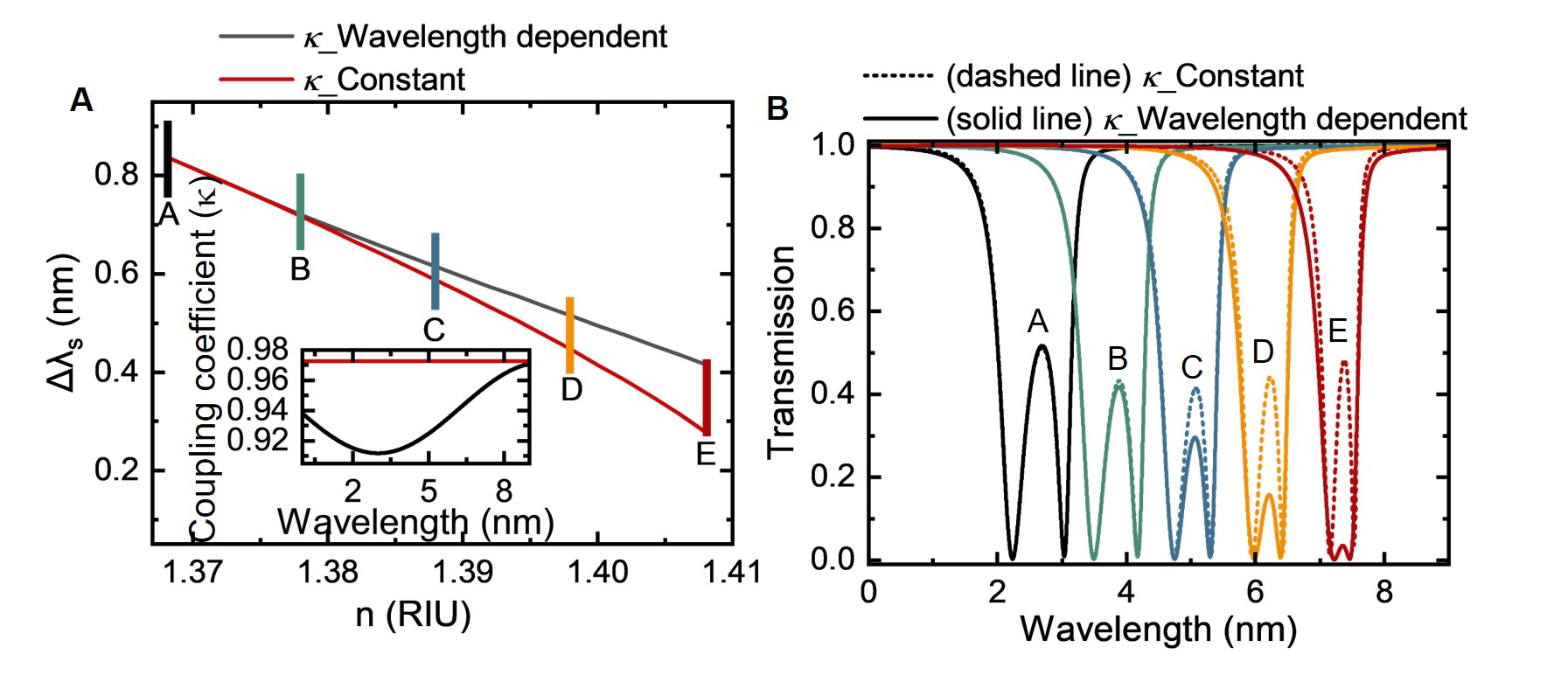 |
| --- |
| Figure S7. The nonlinearity variations of mode splitting. (A) Comparison of mode splitting variation with changing refractive indices for constant and wavelength-dependent coupling coefficients, with the inset showing the values of wavelength-dependent *κ*. (B) Spectral responses of the mode splitting with constant *κ* (dashed line) and wavelength-dependent *κ* (solid line).  S11 The subwavelength grating coupler  The experimental setup employs two on-chip subwavelength grating couplers with a 10° angle for free-space coupling, facilitating light coupling from optical fibers/chip to the chip/optical fibers. The scanning electron microscope (SEM) image of the coupler is shown in Figure S8(A). The measured peak coupling efficiency is −6.24 dB at 1568 nm, with a 3 dB bandwidth of 45 nm, as illustrated in Figure S8(B). These couplers support a wavelength range of 1525–1600 nm, covering the spectral region used in our experiments. Additional details have been incorporated into the supplementary material for completeness.   \| 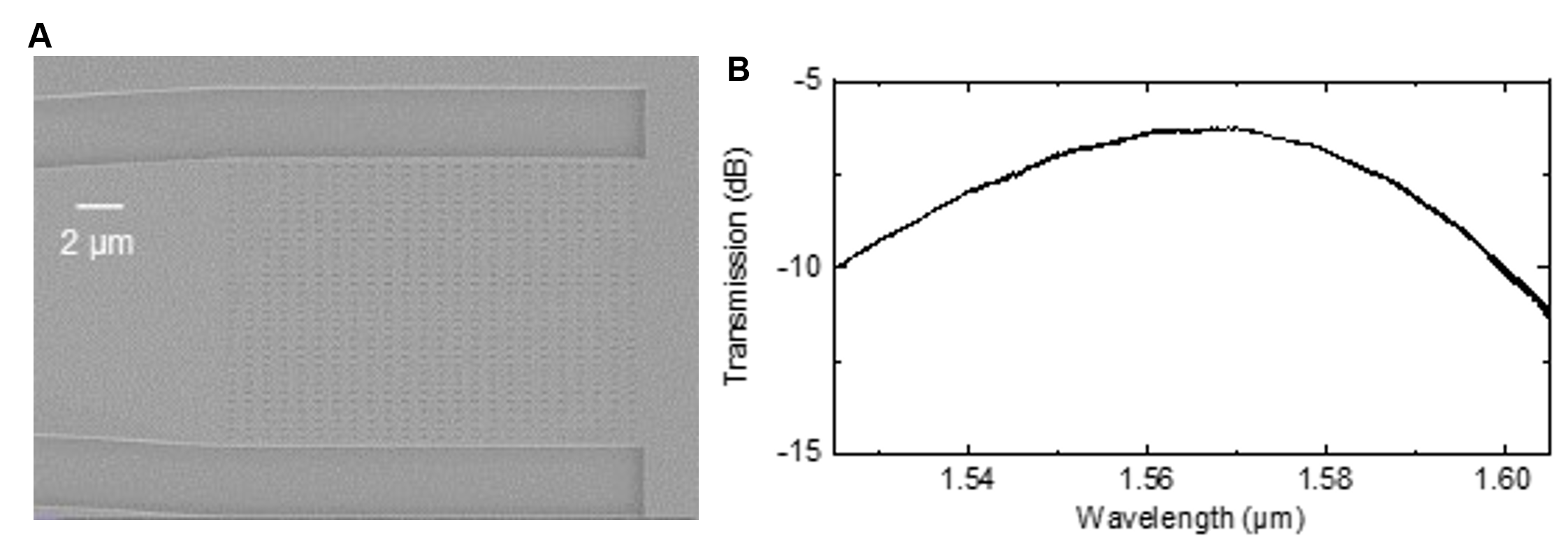 \| \| --- \| \| Figure S8. (A) The SEM figure of the grating coupler. (B) The transmission spectrum of the subwavelength grating coupler. \| |

S12 The sensing performance of the unsolvable splitting mode

The mode broadening of the unresolvable splitting is also a typical method used to detect tiny refractometric perturbations, such as a single particle^[16]^. The spectral responses with normalized intensity of an unresolved resonance during the detection of 0.01 pg/mL streptavidin are shown in Figure S9(A). The linewidth is defined as the range where the normalized spectral intensity is below 0.01. Figure S9(B) shows the sensor's spectra achieving the equilibrium state in PBS washing before and after streptavidin injection. The average linewidth change at the equilibrium state is summarized in Figure S9(C), showing a noticeable reduction in linewidth of 25.66 pm. The initial error bar (standard deviation) is 3.98 pm. According to the LOD definition, the noise limit (3σ≈11.94 pm) is lower than the variation in signal (25.66 pm). Therefore, mode broadening is an effective approach to detect low concentration analytes. In practice, unresolvable splitting is common due to the relatively low *Q* factor of MWMRs, typically ranging from 10³ ~ 10⁴,^[17-19]^ indicating that the mode splitting generally cannot be recognized when the 3 dB bandwidth is below 0.15 nm.

| 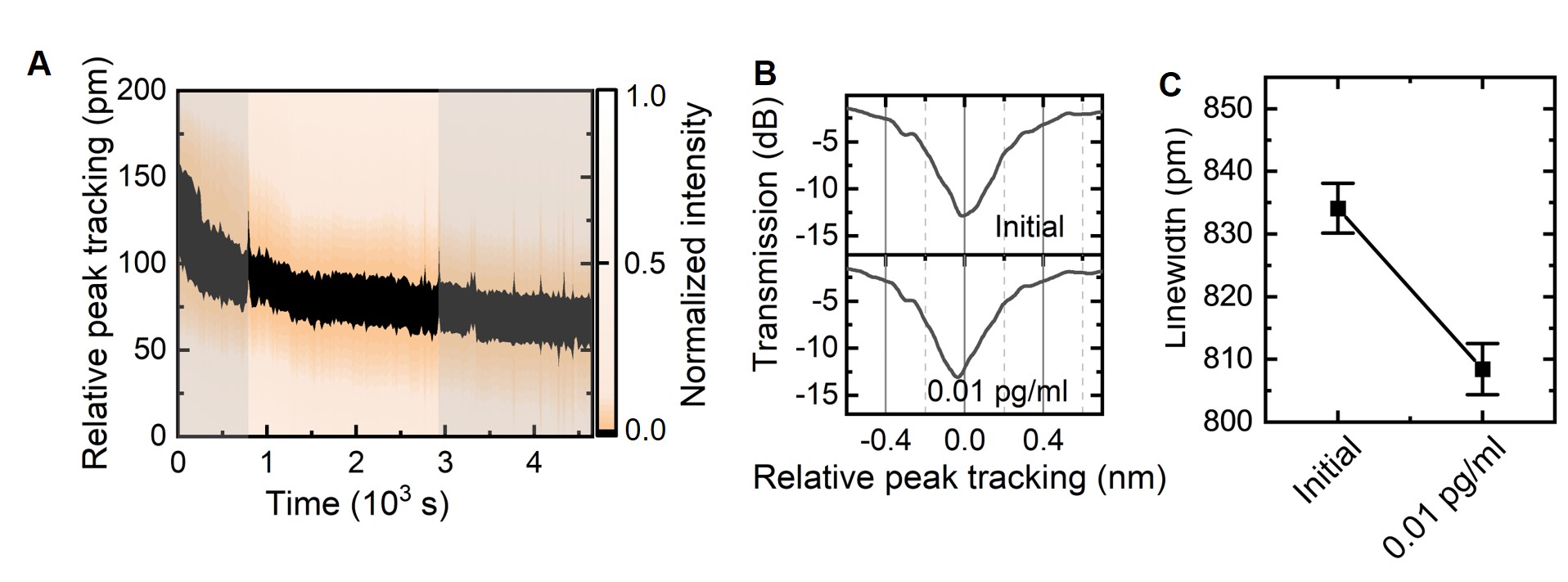 |
| --- |
| Figure S9. The sensing performance of the unresolvable splitting mode. (A) The responses of the unresolvable splitting mode to detecting 0.01 pg/mL streptavidin with normalized intensity. (B) The sensor spectra achieve the equilibrium state in PBS washing before and after streptavidin injection. (C) The summarized linewidth responses to streptavidin detection. |

References

[1] B. E. Little, J. P. Laine, S. T. Chu, *Opt. Lett*. **1997**, 22, 4–6.

[2] J. Zhu, S. K. Ozdemir, Y. F. Xiao, L. Li, L. He, D. R. Chen, L. Yang, *Nat. Photonics* **2009**, 4, 46–49.

[3] Y. Chen, J. Duan, J. Li, X. Xu, J. Wang, *Photonics Res*. **2024**, 12, 1794–1801.

[4] J. Li, W. Li, Y. Feng, J. Wang, Y. Yao, Y. Sun, Y. Zou, F. He, J. Duan, *Nano Lett.* **2024**, 24, 3906–3913.

[5] W. Li, J. Li, L. Yu, Y. Feng, Y. Yao, Y. Sun, Y. Zou, X. Xu, *APL Photonics* **2023**, 8, 016102.

[6] M. de Goede, M. Dijkstra, L. Chang, N. Acharyya, G. Kozyreff, R. Obregon, E. Martinez, S. M. Garcia-Blanco, *Opt. Express* **2021**, 29, 346–358.

[7] N. Acharyya, M. Maher, G. Kozyreff, *Opt. Express* **2019**, 27, 34997–35011.

[8] X. Lu, A. Rao, G. Moille, D. A. Westly, K. Srinivasan, *Photonics Res*. **2020**, 8, 1676–1686.

[9] X. Lu, S. Rogers, W. C. Jiang, Q. Lin, *Appl. Phys. Lett*. **2014**, 105, 151104.

[10] S. Gafsi, J. D. Ryckman, *Laser Photonics Rev*. **2025**, 19, 2401579.

[11] R. Cheng, Y. Han, L. Chrostowski, Opt. Express **2019**, 27, 9516–9535.

[12] I. Teraoka, Opt. Commun. **2015**, 339, 108–114.

[13] B. E. A. Saleh, M. C. Teich, Fundamentals of Photonics, Wiley, Hoboken, NJ, **2019**.

[14] J. Čtyroký, I. Richter, M. Šiňor, Opt. Quantum Electron. **2007**, 38, 781–797.

[15] X. Xu, H. Subbaraman, J. Covey, D. Kwong, A. Hosseini, R. T. Chen, Appl. Phys. Lett. **2012**, 101, 031109.

[16] Y. Hu, L. Shao, S. Arnold, Y. C. Liu, C. Y. Ma, Y. F. Xiao, Phys. Rev. A **2014**, 90, 043847.

[17] C. W. Chang, X. Xu, S. Chakravarty, H. C. Huang, L. W. Tu, Q. Y. Chen, H. Dalir, M. A. Krainak, R. T. Chen, Biosens. Bioelectron. **2019**, 141, 111396.

[18] V. Donzella, A. Sherwali, J. Flueckiger, S. M. Grist, S. T. Fard, L. Chrostowski, Opt. Express **2015**, 23, 4791–4803.

[19] H. Yan, L. Huang, X. Xu, S. Chakravarty, N. Tang, H. Tian, R. T. Chen, Opt. Express **2016**, 24, 29724–29733.
